# Trends in Views of Articles Published in 3 Leading Medical Journals During the COVID-19 Pandemic

**DOI:** 10.1001/jamanetworkopen.2021.6459

**Published:** 2021-04-01

**Authors:** Andrew J. Giustini, Alan R. Schroeder, David M. Axelrod

**Affiliations:** 1 Department of Anesthesiology, Stanford University School of Medicine, Stanford, California; 2 Department of Pediatrics, Stanford University School of Medicine, Stanford, California

## Abstract

This cross-sectional study assesses changes in views of medical scientific articles published in 3 leading medical journals since the start of the COVID-19 pandemic.

## Introduction

The COVID-19 pandemic is changing the peer review, publication, and readership of scientific articles.^1,2,3^ The scientific community has voiced concern that the focus on COVID-19 adversely affects dissemination of research into other diseases.^4,5^ Recently, the number of article views has been recognized as a metric for article impact.^6^ In this study, we sought to assess the trends in views of articles published in 3 leading medical journals during the pandemic.

## Methods

Because no patients were involved in this study (only analysis of journal article reads), we did not obtain institutional review board approval or informed consent. To assess changes in views of medical scientific articles, in this cross-sectional study we examined full and PDF views of articles published by 3 widely read, English-language, general medical journals—*JAMA*,* The New England Journal of Medicine *(*NEJM*), and *BMJ*—from January to July of 2019 and 2020. All articles other than journal mastheads were included in data collection. Article types included research articles, educational articles, opinion, reviews, letters, erratum, and scientific news.

Views data were acquired by inspecting the metrics information for each article provided by the journal websites with the Scrapy web scraping and website parsing package version 2.3.0 (Scrapy) for Python statistical software version 3.8.3 (Python Software Foundation) with the Spyder open-source interface version 4.1.4. We first determined whether articles were COVID-19 focused and original research (yes or no). COVID-19–focused articles were defined as those that referenced COVID-19 (or a synonymous term) in the title, or whose content was judged by the primary author (A.J.G.) to be primarily pandemic related. Unclear article categorization was decided in consensus by all 3 authors. Articles were categorized as original research if they were original research articles, including meta-analyses.

We compared the views of non–COVID-19 original research articles from March 2020 (when COVID-19 attention began to mount) to July 2020 with the same period in 2019. Because of journal variation in metric reporting methods, we standardized view accrual time by summing views through the end of the month following the date of issue. Differences in median views of the 457 relevant articles were assessed with the Wilcoxon rank-sum test using R statistical software version 4.0.2 with the RStudio version 1.3.1073 interface (both from R Project for Statistical Computing). We then performed subgroup analyses on the 3 journals with a Bonferroni correction for multiple comparisons, with significance set at 2-tailed *P* = .017. Data analysis was performed from October to December 2020.

## Results

**Figure 1.**
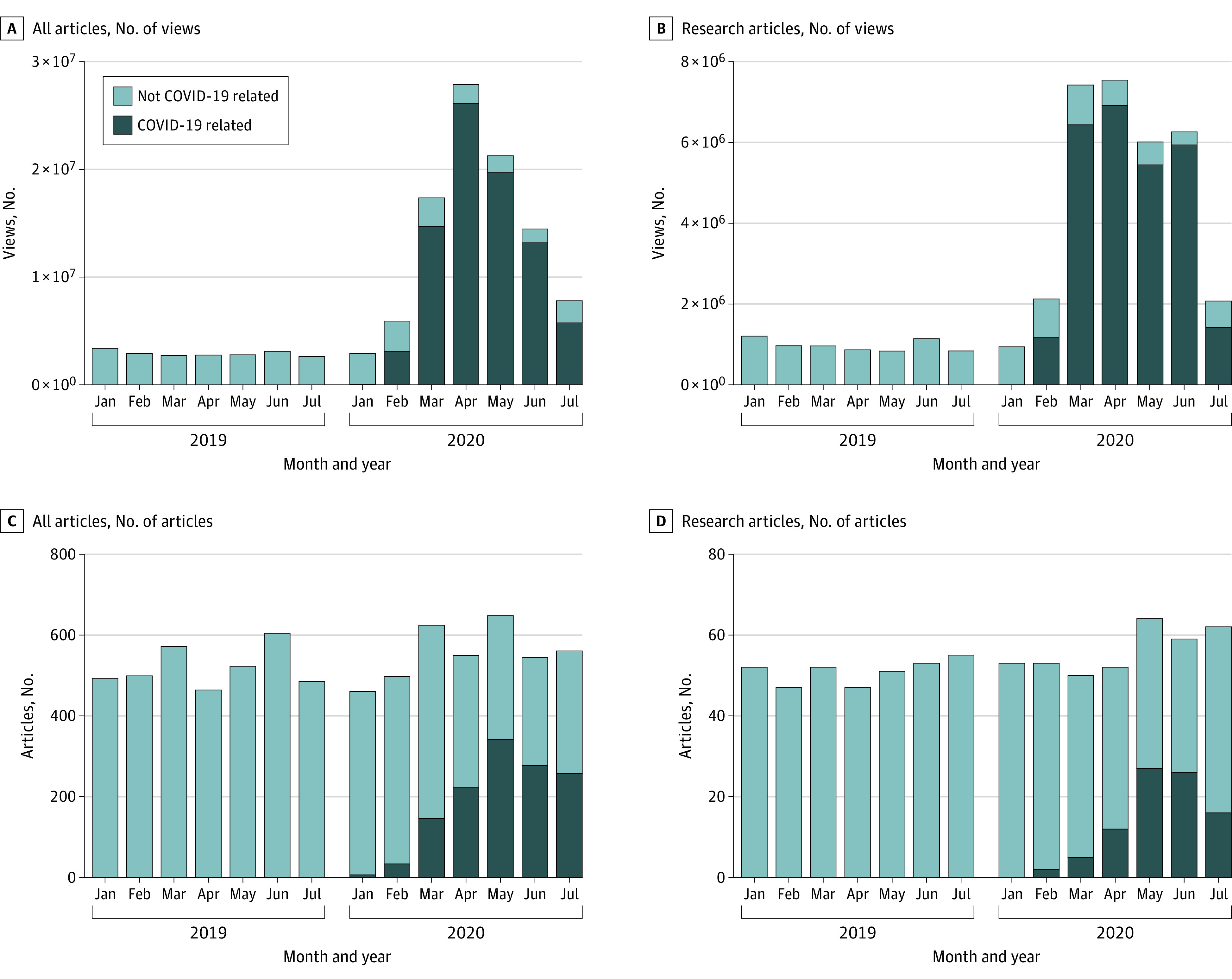
Total Views and Number of Articles Published in *BMJ*, *JAMA*, and *New England Journal of Medicine *(*NEJM*) in January to July of 2019 and 2020 Graphs show number of views for all articles (A) and research articles only (B) and number of articles published for all articles (C) and research articles only (D) in the 3 journals over the same period in 2019 and 2020.

**Figure 2.**
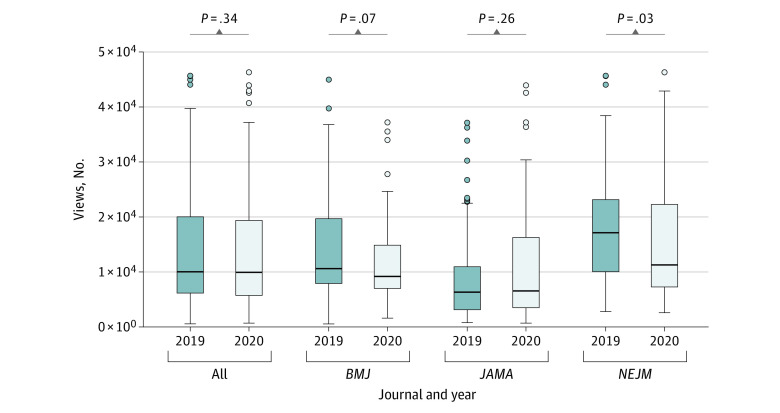
Differences in Numbers of Research Articles Published March to July of 2019 and 2020 in *BMJ*, *JAMA*, and *New England Journal of Medicine *(*NEJM*) Box plots show medians (lines within boxes) and interquartile ranges (bottoms and tops of boxes) of articles published in the 3 journals. Scale is limited to 500 000 to better show boxes. Circles denote outliers. Whiskers denote values within 1.5 times the interquartile range from the upper or lower quartile.

 In total, the number of views for 7528 articles were collected: 4059 articles from *BMJ*, 2079 from *JAMA*, and 1390 from *NEJM*. In March to July of 2020, the median (interquartile range) number of views of COVID-19 original research articles was 117 341.5 (51 114-294 8595.5) views, and the median (interquartile range) number of views of non–COVID-19 original research articles was 10 171 (5848-20 406) views. In March to July 2019, there were 258 non–COVID-19 research articles published (68 in *BMJ*, 97 in *JAMA*, and 93 in *NEJM*), compared with 199 non–COVID-19 original research articles published in March to July 2020 (49 in *BMJ*, 70 in *JAMA*, and 80 in *NEJM*), a decrease of 23%. Overall readership of articles between March to July 2019 and March to July 2020 increased by 557%, whereas the total number of articles published per month remained constant (Figure 1). Although the total number of non–COVID-19 original research articles decreased from 2019 to 2020 (Figure 1B and 1D), the median number of views of each article was not substantially different between March to July of 2019 and March to July 2020 (Figure 2).

## Discussion

The COVID-19 pandemic has increased overall article views for major medical journals in 2020, with unprecedented views per article for COVID-19–related publications. Although the total number of published original non–COVID-19 research articles decreased during the pandemic in these 3 journals, the number of views per article has remained constant, implying that individual non–COVID-19 original research articles are receiving similar attention as before the pandemic. The pandemic may detrimentally affect the broader evidence base because fewer non–COVID-19 research articles have been published in the 3 journals studied. This work begins to address the question of how the COVID-19 pandemic has affected attention to other diseases in the medical literature. These findings may be limited by different approaches to page view reporting and variable numbers of articles published between the studied journals.
